# Time-varying associations between COVID-19 case incidence and community-level sociodemographic, occupational, environmental, and mobility risk factors in Massachusetts

**DOI:** 10.1186/s12879-021-06389-w

**Published:** 2021-07-16

**Authors:** Koen F. Tieskens, Prasad Patil, Jonathan I. Levy, Paige Brochu, Kevin J. Lane, M. Patricia Fabian, Fei Carnes, Beth M. Haley, Keith R. Spangler, Jessica H. Leibler

**Affiliations:** 1 Department of Environmental Health, Boston University School of Public Health, 715 Albany St, Boston, MA 02118 USA; 2 Department of Biostatistics, Boston University School of Public Health, Boston, MA USA

## Abstract

**Background:**

Associations between community-level risk factors and COVID-19 incidence have been used to identify vulnerable subpopulations and target interventions, but the variability of these associations over time remains largely unknown. We evaluated variability in the associations between community-level predictors and COVID-19 case incidence in 351 cities and towns in Massachusetts from March to October 2020.

**Methods:**

Using publicly available sociodemographic, occupational, environmental, and mobility datasets, we developed mixed-effect, adjusted Poisson regression models to depict associations between these variables and town-level COVID-19 case incidence data across five distinct time periods from March to October 2020. We examined town-level demographic variables, including population proportions by race, ethnicity, and age, as well as factors related to occupation, housing density, economic vulnerability, air pollution (PM_2.5_), and institutional facilities. We calculated incidence rate ratios (IRR) associated with these predictors and compared these values across the multiple time periods to assess variability in the observed associations over time.

**Results:**

Associations between key predictor variables and town-level incidence varied across the five time periods. We observed reductions over time in the association with percentage of Black residents (IRR = 1.12 [95%CI: 1.12–1.13]) in early spring, IRR = 1.01 [95%CI: 1.00–1.01] in early fall) and COVID-19 incidence. The association with number of long-term care facility beds per capita also decreased over time (IRR = 1.28 [95%CI: 1.26–1.31] in spring, IRR = 1.07 [95%CI: 1.05–1.09] in fall). Controlling for other factors, towns with higher percentages of essential workers experienced elevated incidences of COVID-19 throughout the pandemic (e.g., IRR = 1.30 [95%CI: 1.27–1.33] in spring, IRR = 1.20 [95%CI: 1.17–1.22] in fall). Towns with higher proportions of Latinx residents also had sustained elevated incidence over time (IRR = 1.19 [95%CI: 1.18–1.21] in spring, IRR = 1.14 [95%CI: 1.13–1.15] in fall).

**Conclusions:**

Town-level COVID-19 risk factors varied with time in this study. In Massachusetts, racial (but not ethnic) disparities in COVID-19 incidence may have decreased across the first 8 months of the pandemic, perhaps indicating greater success in risk mitigation in selected communities. Our approach can be used to evaluate effectiveness of public health interventions and target specific mitigation efforts on the community level.

## Introduction

As of May 2021, the United States had the highest number of Coronavirus Disease 2019 (COVID-19) cases and deaths in the world [1]. Within the US, disease incidence has varied substantially among states with different policy interventions and adherence to public health guidance, [2–4] and there is also significant variability within states [5]. For example, in Massachusetts, during the two-week period from January 10–23, 2021, COVID-19 average daily incidence exceeded 100 confirmed cases per 100,000 persons in multiple urban communities (including Chelsea, Lawrence and New Bedford), with a low of zero in a number of more-rural communities [6].

Early in the pandemic, multiple community-level factors were associated with higher COVID-19 incidence, with disproportionate burdens among communities with more racial and ethnic diversity and workers in essential services [7–10]. A growing literature demonstrates the pandemic’s heightened burden on people experiencing poverty, living in crowded housing, working at jobs without telework options, and/or having reduced access to testing or medical care [11–15]. Higher COVID-19 case incidence is associated with greater percentage of immigrants and lower education at the community level, likely due to occupational, medical, and housing risk factors that elevate risk of disease transmission and severity [16]. Likewise, environmental exposures, including air pollution and reduced green space, are associated with increased case incidence at the community level [17–19].

While this research considering sociodemographic data and COVID-19 cases on the community reinforces the complexity of the sociodemographic and environmental predictors of COVID-19, such studies are primarily cross-sectional in design. As the pandemic evolved, factors associated with elevated case incidence are likely to have evolved as well, given changes in work patterns, behaviors, and state and local policies. To our knowledge, no studies to date have investigated the time-dependent association of COVID-19 incidence at a town level with key predictors.

In this study, we present a novel set of models analyzing associations between publicly available town-level data and COVID-19 incidence, with the goal of evaluating changes in key town-level predictors across Massachusetts over time. We ran parallel regression models with the same set of predictors over five different time periods from the first recorded case in Massachusetts in March 2020 to October 2020, prior to the introduction of vaccines, to assess the temporal variability of the relative magnitude and significance of these predictors on COVID-19 incidence. Characterization of the time-variant nature of these predictors may assist in future efforts to target interventions to specific subpopulations and assess the effectiveness of mitigation strategies on the community level over time.

## Methods

### Time periods

**Fig. 1:**
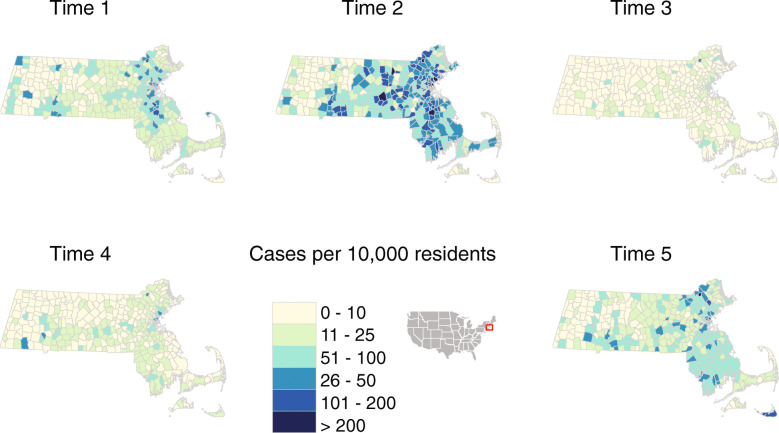
COVID-19 case incidence per 10,000 inhabitants during five phases of the pandemic in Massachusetts

We used publicly available case data for 351 cities and towns published by the Massachusetts Department of Public Health (MA DPH) from April 14, 2020 through October 29, 2020 [20]. Case data were centrally reported to MA DPH by all laboratories across the state that conducted COVID-19 testing, and case counts by town and in aggregate across the state were published weekly. Per MA DPH, COVID-19 cases were defined as PCR-confirmed cases and excluded probable cases (such as those with positive antigen tests only or symptoms consistent with COVID-19 without molecular confirmation) [6]. We pulled case data from the MA DPH website in November 2020 that included any changes or updates to case counts made by that time. We stratified that case data across 5 time periods selected to reflect distinct waves of case incidence in the state, starting with the spring “first wave” (March 2 – April 14, 2020, and April 15 – June 3), the summer nadir (June 4 – July 15, 2020, and July 16 – September 2, 2020), and the early months of the “second wave” in the fall (September 3 – October 29, 2020). The specific end date was chosen based on COVID-19 case incidence data availability at the time of study. Figure 1 shows case incidence per 10,000 inhabitants per town in Massachusetts for each time period.

### Data sources

Table 1 lists the sociodemographic, occupational, environmental, and mobility datasets at the city/town level that were integrated with data on COVID-19 incidence during the five time periods. Sociodemographic, occupational, and economic data were extracted from the 2014–2018 American Community Survey (ACS) 5-year estimates at the census tract level. Data were scaled to town level by downloading ACS data at the census tract level in absolute numbers, aggregating to town level, and proportions calculated with town population estimates [21]. While most towns consist of one or more census tracts, a small set of rural census tracts contained several smaller towns, yielding identical ACS percentage values for these towns. Estimates for percentage of essential workers were modified from ACS data based on an integrated dataset developed by the American Civil Liberties Union, which restricted ACS data on service workers to those job types considered “essential” during the pandemic including healthcare practitioners, transportation occupation, food preparation, etc. [22] Data on town-level imprisoned population were obtained through MassGIS [23] and long-term care facility beds by town were obtained from MA DPH [20, 24, 25]. Housing density data was evaluated as percentage of residences with 1, 1.5 and 2 persons per room per ACS. The percentage of people commuting to work was estimated using the SafeGraph Social Distancing Metrics dataset derived from anonymized cell phone mobility data, where a commuter is defined as someone who spends 8 hours (full-time) or 4 hours (part-time) a day from Monday-Friday in a single location away from their home census block group [26]. For each town, we aggregated the percentage of people working full-time or part-time during each individual time period using a one-week lag to include the effects of increased frequency of working from home. Annual fine particulate matter (PM_2.5_) concentrations from 2015 were obtained from Kloog et al. at a 1-km grid resolution, averaged by town to reflect a general measure of air pollution [27]. Population density, as a proxy for local transmission risk, was calculated per town using 100 m cell size population grids derived from the 2010 Census counts available at the census block level (assuming equal spatial distribution of people within census blocks). For each town we averaged the cell values depicting population totals in each cell at different Euclidean distance radii (5, 20, 50, and 100 km) to capture the effects of urbanicity and population density at different scales [28].

**Table 1:** Sociodemographic, occupational, environmental, and mobility risk factors used to predict COVID-19 case incidence in cities and towns of Massachusetts

Variable name	Description
% Racial minorities	Percentage of non-white people^a^
% Black	Percentage of people identifying as African American/ Black^a^
% Hispanic/Latinx	Percentage of people identifying as Latino/Hispanic^a^
% Age > 80	Percentage of people older than 80 years^a^
% Age > 70	Percentage of people older than 70 years^a^
% Age < 20	Percentage of people younger than 20 years^a^
% Below poverty	Percentage of people living below poverty line^a^
% Disabilities	Percentage of people with disability^a^
% Essential services	Percentage of people with essential service job^a^
% No health insurance	Percentage of people without health insurance coverage^a^
% More than 2 per room	Percentage of housing units with occupancy > 2 persons per room^a^
% More than 1.5 per room	Percentage of housing units with occupancy > 1.5 persons per room^a^
% More than 1 per room	Percentage of housing units with occupancy > 1 persons per room^a^
% Public transportation	Percentage of people traveling to work by public transportation^a^
% Undergraduates	Percentage of people enrolled in undergraduate higher education^a^
Prison population	Percentage of incarcerated population^b^
LTCF (# beds)	Number of long term care facility beds as percentage of population^c^
PM_2.5_	Mean annual PM_2.5_ level^d^
Population density (5 km)	Number of people living within 5 km radius from town^a^
Population density (20 km)	Number of people living within 20 km radius from town^a^
Population density (50 km)	Number of people living within 50 km radius from town^a^
Population density (100 km)	Number of people living within 100 km radius from town^a^
% Going to work	Estimated percentage of population going to work outside home^e^

^a^ Data derived from the 2014–2018 American Community Survey^21^

^b^ Data derived from MassGIS^23^

^c^ Data derived from the Massachusetts Department of Public Health^20^

^d^ Data derived from Kloog et al. 2014^27^

^e^ Data derived from SafeGraph mobility datasets^26^

### Statistical analysis

**Fig. 2:**
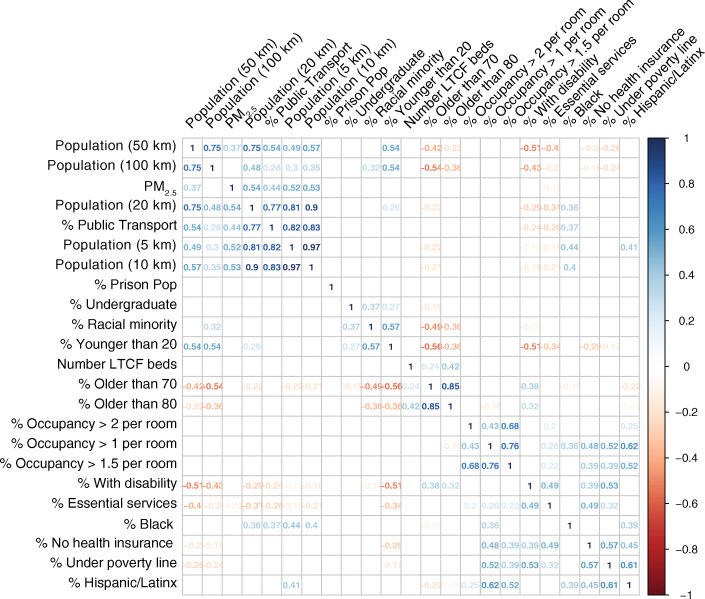
Pearson correlation coefficients for bivariate combinations of predictor variables (only showing correlations where *p* < 0.001)

We developed a series of mixed-effect Poisson regression models to predict COVID-19 case incidence by town for each of the five distinct time periods of the pandemic. We used the most recent population estimate of each town as an offset to account for differences in population per town [29]. To minimize multi-collinearity while maintaining a good fit, we used a backward selection process of covariates where we stepwise excluded covariates with a variance inflation factor higher than 2.5 in the regression of the first time period [30]. All numerical predictors were normalized to zero mean and standard deviation of 1. We included the county of each town as a random effect to control for spatial autocorrelation of residuals (351 towns divided over 14 counties). For every period except the first period ending on April 14, we included an ordinal variable of the COVID-19 case count per capita of the previous period, divided into quintiles, as a random effect to adjust for between-period temporal autocorrelation. To assess collinearity structure of the predictor variables, we calculated Pearson correlations between all bivariate predictor combinations and presented them in a correlation matrix (Fig. 2). Mixed-effect models were executed in R 3.6.1 [31] using the *glmer* function from the *lme4* package [32].

## Results

The backward predictor selection process resulted in a set of eight fixed-effect predictor variables that had a good fit in all 5 phases of the pandemic between the beginning of March 2020 and the end of October 2020. Excluded variables either caused high levels of multi-collinearity or had no significant association with the response variable. The correlation matrix in Fig. 2 shows a cluster of population density variables with public transportation and PM_2.5_ in the upper left corner and a cluster of housing crowdedness with percent of the population working in essential services, under the poverty line, or with disabilities. Both clusters show positive correlations with % Black population and Hispanic/Latino (henceforth referred to as Latinx) population. Point estimates and associated 95% confidence intervals are presented for all eight predictors in the model across all time periods (Table 2).

**Table 2:** Select associations between sociodemographic and economic predictors and COVID-19 incidence by time period

Variable^c^	Incidence rate ratio (95% CI)^a^				
	Time 1^b^	Time 2	Time 3	Time 4	Time 5
% Age > 80	1.04 (1.02–1.07)	1.14 (1.12–1.16)	1.03 (0.98–1.08)	1.05 (1.01–1.09)	1.03 (1.00–1.05)
% Black	1.12 (1.12–1.13)	1.10 (1.09–1.1)	1.06 (1.04–1.07)	1.05 (1.04–1.06)	1.01 (1.00–1.01)
% Hispanic/Latinx	1.19 (1.18–1.21)	1.13 (1.12–1.14)	1.15 (1.13–1.17)	1.07 (1.06–1.09)	1.14 (1.13–1.15)
% No health insurance	0.85 (0.84–0.87)	1.09 (1.08–1.11)	1.14 (1.09–1.18)	1.21 (1.18–1.25)	1.06 (1.04–1.08)
% Essential services	1.30 (1.27–1.33)	1.21 (1.19–1.23)	1.20 (1.15–1.25)	1.24 (1.2–1.28)	1.20 (1.17–1.22)
LTCF beds per capita	1.28 (1.26–1.31)	1.23 (1.21–1.24)	1.09 (1.05–1.13)	1.10 (1.07–1.13)	1.07 (1.05–1.09)
Population (20 km)	1.21 (1.19–1.23)	0.98 (0.97–0.99)	1.01 (0.98–1.05)	1.23 (1.2–1.26)	0.98 (0.96–1)
% Undergraduates	1.02 (1.01–1.04)	0.98 (0.97–0.99)	0.96 (0.93–1)	0.97 (0.95–0.99)	1.06 (1.04–1.07)

^a^ Odds ratios generated through mixed-effect multivariable Poisson regression. Models were adjusted for all other covariates presented here

^b^ Dates for study periods: Time 1: before April 14; Time 2: April 15 – June 3; Time 3: June 4 – July 15; Time 4: July 16 – September 2; Time 5: September 3 – October 29

^c^ Variables reflect percent of the town population by given characteristic, so percent of town population > 80 years or the percent of the town population Black/African American, for example. Data generated from the 2014–2018 American Community Survey^21^

**Fig. 3:**
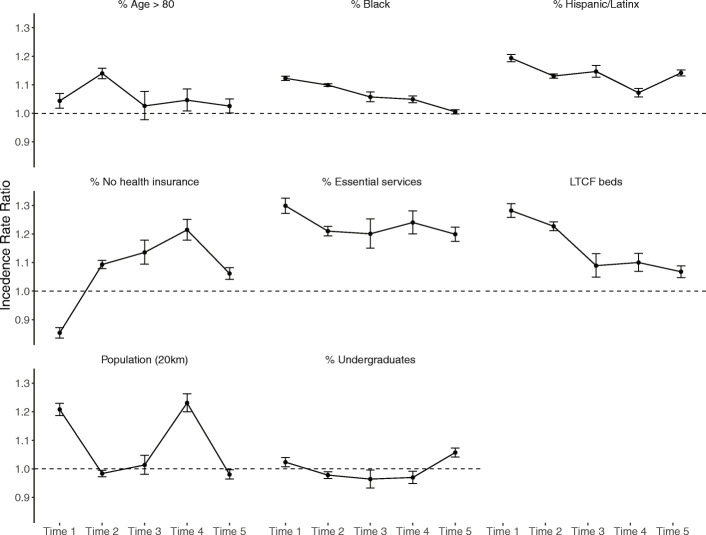
Temporal change of log odds (with 95% confidence intervals) of mixed effects models predicting the number of COVID-19 cases per town weighted by population during five phases of the pandemic in Massachusetts

Of the final selection of predictors, almost all showed notable variations in association with COVID-19 cases over the study period (Fig. 3), which we assessed descriptively and visually across the five time periods. Whereas an increase of one standard deviation in the percentage of town residents identifying as Black was associated with an increase of more than 10% in COVID-19 cases before April 14, the association steadily decreased and became statistically non-significant by September–October. At the start of the pandemic, we observed a nearly 20% increase in COVID-19 cases by town associated with one standard deviation increase in the percentage of town residents identifying as Latinx, but the strength of this positive association varied over time, with a decreasing association from April to September and an uptick in the association in the last phase of our model (September 3rd to October 29th).

We observed a decreasing trend in the association between percent of the town population older than 80 years and the number of long-term care facility beds per town per capita with COVID-19 incidence, indicating that the presence of older residents and institutional care facilities in a town had a stronger association with incidence in the early, but not the later, periods of the study. The percent of essential workers by town consistently showed a significant positive association with COVID-19 incidence throughout all time periods.

Population density, measured as the total number of people living within 20 km from the boundary of each town, showed notable changes in association with COVID-19 case incidence, with high positive associations in the first and fourth time periods, and non-significant associations during the other time periods. The undergraduate student population, measured as the percentage of the town population enrolled in undergraduate education, was associated with a small positive effect on COVID-19 incidence early in the pandemic and a larger association after September 3rd, when several universities throughout the state resumed in-person or hybrid classes after shutting down for in-person learning in March or April 2020.

## Discussion

Our findings illustrate the time-varying nature of sociodemographic and economic predictors of COVID-19 case incidence at the community level in Massachusetts during an eight-month period. These observations suggest that fixed assumptions regarding community-level COVID-19 vulnerability, such as increased risk among Black communities or continued elevated relative risk among the elderly, may not accurately represent the pandemic at every point in time, and that these associations should be continually reassessed for relevance alongside shifting mitigation efforts, policies, and individual behavior. While other studies have identified racial/ethnic disparities in COVID-19 incidence in Massachusetts and the extent to which these patterns are explained by social factors [7], our study provides novel insight about how both patterns and predictors change over time. Likewise, associations that remain significant over time with consistent coefficients in these adjusted models, such as workers in essential services, may suggest that existing approaches to reduce risk within these subgroups are less effective, perhaps due to structural challenges in reducing certain exposures.

We note that the community-level predictor variables used here serve as proxies for underlying factors that influence individual viral exposure risk. This distinction is important in our analysis, as it remains possible that analyses using individual-level predictors would lead to different findings. However, our analysis provides insight into sociodemographic patterns of COVID-19 and subpopulations who may be at elevated risk, informing community-scale public health interventions, and vaccination strategies by location, as well as a useful and adaptable structure to assess risk alongside public data.

The consistently elevated risk observed in communities with increased Latinx populations (in models adjusted for essential workers and other sociodemographic variables) may underscore challenges in reaching these communities with successful interventions, or barriers to reduced exposure in these communities. The sustained elevated risk of Latinx populations throughout the first 8 months of the pandemic is consistent with recent studies [7, 33]. In our models, Latinx population was positively correlated with greater housing density, suggesting that this ethnicity variable may serve in part as a proxy for crowded housing (> 1 person/room) in our models. Within-household transmission is an established risk factor for COVID-19 transmission [34, 35], and it is possible that our findings here reflect established challenges in reducing transmission within housing environments where residents are unable to meaningfully distance from an infectious individual.

We did not observe a correlation between town-level housing density and Black populations, or between this race variable and other sociodemographic covariates in our model. This finding suggests that other factors beyond the scope of our analysis may be responsible for the elevated COVID-19 risks faced by towns with higher percentage of Black residents, especially early in the pandemic. Our findings parallel core conclusions of Figueroa et al., who evaluated cross-sectional COVID-19 case incidence alongside demographic data in Massachusetts from March–May 2020 [7]. The authors noted racial disparity in disease incidence in the early wave after adjustment for essential workers, immigration, and household size, while the association between COVID-19 cases and Latinx population was attenuated in models adjusted for these factors. Together, our studies support the hypotheses that systemic racism and inequities not otherwise captured in core demographic datasets play a role in driving COVID-19 racial inequities and that distinct analyses focused on systemic inequities are needed to fully understand the specific risks faced by Black individuals and communities.

Reduced risk of COVID-19 in communities with higher percentages of Black residents, adjusting for other factors, may suggest that the clear racial disparity observed in early months of the pandemic has diminished over time in Massachusetts [5, 10, 36, 37]. Our finding parallels other observations as to the reduced racial disparity in COVID-19 cases over time [38]. Likewise, substantial reductions in the association between long-term care beds and percentage of town population over 80 years and COVID-19 incidence may reflect success in interventions to protect the elderly, especially those living in long-term or nursing care facilities, after the initial, devastating impacts on this population early in the pandemic [39, 40]. It remains possible that factors not included in our analysis, notably biases associated with testing availability or other unstudied correlates, are responsible for the reductions in risk we observe here, especially by race. Analysis of more severe outcomes, including hospitalization and deaths, would help inform these questions, although requiring less-nuanced temporal resolution, and should be prioritized for future work.

The persistent positive association between essential workers and COVID-19 may reflect the continued vulnerability of this workforce to viral infection, despite workplace controls and personal mitigation behaviors, such as masking and maintaining social distance. Essential workers remained at the highest risk of all subgroups studied in our models throughout the pandemic, highlighting key challenges in protecting these populations, even many months into the pandemic. Interestingly, however, a covariate representing people spending more than 3 h in a location other than their home during office hours showed a null association with COVID-19 cases. The non-significant effect of worker mobility on case incidence may reflect limitations in the underlying data. SafeGraph data includes only 10% of cellphones in the US [41], although the data are highly correlated with true Census population [42]. However, it could also indicate that changes in worker mobility (e.g. “return to work” efforts) were not associated with increased cases when these workers were not part of the essential workforce. This would reinforce that communities with more non-essential workers face distinctly lower risk than those with more essential workers, even with resumption of economic activities, highlighting inequities in exposure profiles on the job.

The significant association between the percentage of town residents without health insurance and case incidence in the spring and summer periods may suggest growing case incidence among immigrants during those times. Due to a state health insurance mandate, the number of non-insured individuals in Massachusetts is low (2.8% of the total state population), but some municipalities have uninsured rates up to 25%, such as Chelsea, Everett, and Lawrence. These communities have higher proportions of younger adults, non-US citizens, and those who are less educated than the insured population [43]. Targeted interventions that focus on communities with elevated percentages of uninsured persons are important in fully understanding disease dynamics in the state.

As noted, our study is limited by the use of town-level covariates, which reduces our ability to draw causal inferences on the individual level; nonetheless, our insights remain relevant for targeted public health strategies. While the use of static predictors from the ACS and other databases allowed us to evaluate and interpret changes in coefficient magnitude and significance over time, it gave us limited ability to capture time-varying exposure factors (with the exception of SafeGraph data). While most of these predictors are expected to be fairly stable during the study period, notably demographic data, it is possible that real-time changes in these covariates may have altered our findings.

It is possible that disparities in testing availability, as noted earlier, contributed to observed trends in case incidence if communities with greater racial diversity had reduced access to case identification, and this is an important area for additional work. It is also possible that time lags in case reporting may have resulted in misclassification in case data by time period, or that incorrect or incomplete address information misidentified town of residence of cases. While we suspect that misclassification on the basis of wrongly attributed case information was largely due to human error and therefore nondifferential, it remains possible that bias affected our estimates in ways that are difficult to predict. In addition, our empirical findings may not generalize to later time periods when vaccines became widely available, although our statistical approach could be directly applied to these time periods and could yield insight regarding how vaccination patterns influenced the sociodemographic predictors of COVID-19 cases.

Our analyses demonstrate that the relevance and magnitude of community-level risk factors for COVID-19 are alterable, suggesting the relative effectiveness of intervention and mitigation efforts by population subgroups (or, conversely, factors that contribute to elevated risk varying over time). Our study highlights the need for local jurisdictions to use up-to-date data on vulnerable and high-risk populations to direct COVID-19 interventions, including vaccinations, rather than data from early in the pandemic. Our models were derived entirely from publicly available data and could be rapidly refit to newer data. Community-level analyses can help characterize social inequities embedded in the pandemic and track the evolution of these inequities with time, highlighting successes as well as disproportionate burdens experienced by vulnerable populations.

## Data Availability

The datasets used during the current study are derived from publicly available data. Compiled datasets are available from the corresponding author on reasonable request.
